# Prevention of post-concussion-like symptoms in patients presenting at the emergency room, early single eye movement desensitization, and reprocessing intervention versus usual care: study protocol for a two-center randomized controlled trial

**DOI:** 10.1186/s13063-018-2902-2

**Published:** 2018-10-12

**Authors:** Cédric Gil-Jardiné, Samantha Al Joboory, Juliane Tortes Saint Jammes, Guillaume Durand, Régis Ribéreau-Gayon, Michel Galinski, Louis-Rachid Salmi, Philippe Revel, Cyril Alexandre Régis, Guillaume Valdenaire, Emmanuel Poulet, Karim Tazarourte, Emmanuel Lagarde

**Affiliations:** 1 University Hospital of Bordeaux, Pole of Emergency Medicine, Bordeaux, France; 2 INSERM, ISPED, Bordeaux Population Health research center INSERM U1219 - “Injury Epidemiology Transport Occupation” team, Bordeaux Cedex, France; 3 CASPERTT, Hospital Center of Cadillac, Lormont, France; 4 Department of Emergency Medicine, University Hospital Edouard Herriot, Hospices civils de Lyon, Lyon, France; 5 University Hospital, Claude Bernard University, Lyon, France; 6 University Hospital of Bordeaux, Pole of Medicine, Bordeaux, France; 7 University Hospital of Bordeaux, Pole of Public Health, Bordeaux, France; 8 Department of Psychiatry, University Hospital Edouard Herriot, Hospices civils de Lyon, Lyon, France; 9 EA 7425 Hesper University Hospital, Claude Bernard University, Lyon, France; 10 Emergency Department, University Hospital of Bordeaux, Pellegrin Hospital, Place Amélie Raba-Léon, 33000 Bordeaux, France; 11 Bordeaux University Hospital, Centre Hospitalier Universitaire de Bordeaux, 12 rue Dubernat, 33400 Talence, France

**Keywords:** Stress, Emergency department, Eye movement desensitization and reprocessing, Post-concussion-like symptoms, Post-traumatic stress disorder, Clinical trial

## Abstract

**Background:**

Recent data suggest that 10–20% of injury patients will suffer for several months after the event from diverse symptoms, generally referred to as post-concussion-like symptoms (PCLS), which will lead to a decline in quality of life. A preliminary randomized control trial suggested that this condition may be induced by the stress experienced during the event or emergency room (ER) stay and can be prevented in up to 75% of patients with a single, early, short eye movement desensitization and reprocessing (EMDR) psychotherapeutic session delivered in the ER.

The protocol of the SOFTER 3 study was designed to compare the impact on 3-month PCLS of early EMDR intervention and usual care in patients presenting at the ER. Secondary outcomes included 3-month post-traumatic stress disorder, 12-month PCLS, self-reported stress at the ER, self-assessed recovery expectation at discharge and 3 months, and self-reported chronic pain at discharge and 3 months.

**Methods:**

This is a two-group, open-label, multicenter, comparative, randomized controlled trial with 3- and 12-month phone follow-up for reports of persisting symptoms (PCLS and post-traumatic stress disorder). Those eligible for inclusion were adults (≥18 years old) presenting at the ER departments of the University Hospital of Bordeaux and University Hospital of Lyon, assessed as being at high risk of PCLS using a three-item scoring rule. The intervention groups were a (1) EMDR Recent Traumatic Episode Protocol intervention performed by a trained psychologist during ER stay or (2) usual care. The number of patients to be enrolled in each group was 223 to evidence a 15% decrease in PCLS prevalence in the EMDR group.

**Discussion:**

In 2012, the year of the last national survey in France, 10.6 million people attended the ER, some of whom did so several times since 18 million visits were recorded in the same year. The SOFTER 3 study therefore addresses a major public health challenge.

**Trial registration:**

Clinical Trials. NCT03400813. Registered 17 January 2018 – retrospectively registered.

**Electronic supplementary material:**

The online version of this article (10.1186/s13063-018-2902-2) contains supplementary material, which is available to authorized users.

## Background

In 2012, when the latest national survey was conducted in France, 10.6 million people reported having attended the emergency room (ER), some of whom did so several times since 18 million ER visits were recorded in the same year [1]. In general, over 90% of those attending the ER will be discharged within hours, without hospitalization [2].

Recent consistent observations [3–6] that 10–20% of injury patients will suffer for several months after the event from diverse symptoms, with a subsequent decline in quality of life that can be significant and delay or prevent the resumption of school or work activities, as well as changing social and family relationships, are of major public health consequences. Approximately 2 million people each year in France are confronted by difficulties of varying degrees whose cause is often unidentified and unrelated to the traumatic event. This link is all the more difficult to make as these symptoms appear to be non-specific, and include headaches, concentration disorders, memory problems, stress intolerance, personality change, and irritability. These symptoms have been described for more than 50 years in the context of head trauma, and were therefore referred to as post-concussion syndrome (PCS). Surprisingly, the most recent results show that these symptoms are not specific to brain injuries and can occur in other patients presenting to the ER [5, 7, 8], greatly expanding the size of the population concerned. In a cross-sectional, observational study of 31,958 high school athletes, Iverson et al. [9] also found that 19% of uninjured boys and 28% of uninjured girls reported having a symptom burden resembling an ICD-10 diagnosis of PCS; thereafter, these symptoms were frequently described as post-concussion-like symptoms (PCLS).

Recognizing that brain damage is not the main cause of these symptoms, researchers have compared patients with and without PCLS with two objectives, namely to predict their occurrence and to understand why they occur. This framework led to the major conclusion that psychological vulnerability, on the one hand, and stress experienced during and in the aftermath of the event, on the other, are the two best predictors of these lasting symptoms. This finding has been repeatedly observed in studies that assess the factors associated with PCLS [9–15].

The study of post-traumatic stress disorder (PTSD) has received renewed interest in view of the psychological pain of soldiers from Western countries returning from overseas following medical trauma, shedding light on this major public health phenomenon also affecting patients who have suffered an accident, physical assault, or an acute medical condition and whose general health remains precarious several months or years later. These studies have led to a better characterization of PTSD, including the individualization of four dimensional components, namely re-experiencing, avoidance, hyperactivation of the nervous system, and cognitive and emotional numbing [16]. Symptoms of PCLS are very similar and even sometimes exactly the same as the last two dimensions of PTSD (hyperactivation of the nervous system and cognitive and emotional numbing). This led various authors to hypothesize that PCLS and PTSD partly share the same causal pathway, in which stress plays a key role. This would be particularly relevant for prevention of PCLS, in particular because, in contrast to PTSD studies, PCLS studies include insufficient numbers and are of low quality to identify credible modes of intervention [17].

Our research team has conducted two studies in the past 10 years that enabled us to further our understanding of PCLS and to look for prevention opportunities. In 2007, we conducted a cohort study of 2018 patients with mild traumatic brain injuries and 1447 other injury patients recruited in the adult ER of Bordeaux University Hospital (Pericles project) [8, 10]. Follow-up to 12 months provided an unprecedented database allowing for in-depth comparisons of patient subgroups. It was this study that showed that PCS, despite its name, was not specific to head trauma [8], and highlighted the importance of stress and the overlap between PCS and PTSD [8, 10]. The data obtained allows us today to compare the performance of risk assessment tools designed to select patients at increased risk of PCS from variables measured in the ER. This last point is of major importance in the preparation of this protocol.

Following the Pericles project, we conducted a pilot study to identify the factors explaining the persistence of symptoms 3 months after an injury event. The key result of this pilot study (manuscript submitted) was that the stress level reported by patients at the end of their ER stay was a powerful predictor of PCLS and PTSD, irrespective of the stress level reported on entering the ER. This important result prompted us to consider testing the feasibility and then the effectiveness of stress management interventions during an ER stay, in the hope of improving the outcomes of traumatized patients.

Results from the literature and these two studies led us to initiate a literature search for the best intervention candidates that would have the potential to lower stress levels during an ER stay.

One of the first ideas proposed for patients who experience a stressful event was to initiate a stress management procedure before the consolidation of stressful memories. This is partly why the practice of psychological debriefing, which consists of debriefing sessions conducted 2–10 days after the critical incident, has been widely disseminated. However, several critical reviews [18] and a Cochrane review [19] have concluded that this form of intervention leads to an increased rate of PTSD.

More promisingly, early exposure therapy, which is based on the extinction of fear through engagement with traumatic memories and clues, appears to be an effective treatment of PTSD [20, 21]. PTSD syndrome can be interpreted as a failure of recovery caused, in part, by failure of the extinction of trauma [22]. This is supported by research conducted on animals showing that early extinction has the potential to alter the consolidation of memory of original fear [23–25]. Rothbaum et al. [18] were the first to show the effectiveness of an extinction-type intervention (prolonged exposure) beginning in the ER in the prevention of PTSD in a sample of 137 patients randomized to three groups. The intervention also included two other sessions 1 and 2 weeks later. The same authors showed that such short-term intervention could also lower PTSD risk in patients with genes previously found to be associated with stress response [26]. Trauma-focused cognitive behavioral therapy delivered within weeks of a potentially traumatic event for people showing signs of distress was also effective in the treatment of acute stress and early PTSD symptoms, and in the prevention of PTSD [27–31].

However, the psychotherapeutic intervention that has thus far proven superior to all other methods is eye movement desensitization and reprocessing (EMDR). Conceived by Francine Shapiro [32], EMDR is an empirically validated psychotherapeutic approach that can rapidly process disturbing experiences adaptively together with the aid of eye movements or other forms of bi-lateral stimulation. Several meta-analyses and Cochrane reviews have shown that this is one of the most effective treatments for PTSD [32–35]. Treatment may be started soon after the trauma, but most often after a complaint from the patient who is already suffering from PTSD symptoms. More recently, a study by Tarquinio et al. [36] showed the effectiveness of an EMDR-based intervention initiated in the first 48 h. The target population of this study was workers who have suffered professional violence (assaults, robberies, etc.).

A study conducted in Israel showed very promising results with a single-session, early modified EMDR session provided in a general hospital inpatient and outpatient setting to 86 patients with acute stress syndrome suffering from intrusion distress following accidents and terrorist bombing attacks [37]. Half of the patients reported immediate fading of intrusive symptoms and general alleviation of distress, 27% described partial alleviation of their symptoms and distress, while 23% reported no improvement. At the 4- and 6-month follow-up, the immediate responders in the terror victims group remained symptom free, while the non-responders endorsed more risk factors for PTSD. These results support other anecdotal reports on the rapid effects of brief EMDR intervention on intrusive symptoms in early uncomplicated post-traumatic cases.

Following the recognition of the failure of psychological debriefing, the issue of difficult access to patients with high levels of stress or dissociation was raised. This was all the more critical as it was known that dissociation at the time at which exposure therapy starts was associated with a poorer response [18]. In response to this challenge and to the increasing number of patients in need of care after manmade catastrophes such as bomb attacks, modified EMDR procedures and protocols adapted for early intervention have been developed to help victims and can be applied soon after trauma, including the emergency response procedure (ERP) [38] and the recent traumatic episode protocol (R-TEP) [39, 40].

The ERP is a short procedure implemented according to procedures designed and tested in emergency contexts, including the ER [40, 41]. The individuals who arrive at the ER show a wide range of disturbance. The greatest benefit of the ERP intervention is expected for patients in a ‘highly agitated’ state (scoring 7–10/10 on the Subjective Units of Disturbance scale, where 0 = no disturbance and 10 = the highest disturbance possible) to those who have moved into a ‘silent terror’ (scoring 10+/10 on the Subjective Units of Disturbance scale).

The R-TEP is an early EMDR current trauma-focused intervention that incorporates and extends the main ideas of the original Recent Event Protocol guidelines first described by Shapiro and Laub in 2008 [42].

The ICD-10 established a set of diagnostic criteria for PCS. In order to meet these criteria, a patient must have had a head injury *“usually sufficiently severe to result in loss of consciousness*” followed by the development, within 4 weeks, of at least three of the eight following symptoms: headache, dizziness, fatigue, irritability, sleep problems, concentration problems, memory problems, and problems tolerating stress. There is relatively little systematic research on the prevention and treatment of PCS [43–46]. A systematic review published in 2010 [45] suggested that cognitive behavioral therapy may be effective in the treatment of PCS. However, the authors found no quality studies and call for more rigorous trials of cognitive behavioral therapy for post-concussion symptoms. Other strategies include information, education and reassurance [47–49]. An emerging literature points to the independent impact of expectations and coping on chronic conditions following trauma, in particular for patients with whiplash and low back pain [50–55]. Reassurance, as provided in the context of cancer [50], low back pain [51, 52], and mild head trauma [47, 49], was found to help patients in their recovery process. It is therefore possible that at least a subgroup of patients who experienced a traumatic injury may benefit from such intervention.

Available research data, both from our studies and that available in the literature, led us to select the EMDR R-TEP procedure. This choice was based on the following considerations:

1. The absence of sufficient literature related to preventive interventions for PCLS
2. The partial overlap between PCLS and PTSD
3. The results of our preliminary studies strongly suggesting that stress plays a major role in PCLS
4. The consensus for the use of EMDR in early prevention of PTSD
5. The growing evidence of a significant psychological component to persistent complaints
6. The failure of early psychological debriefing to prevent PTSD

We then conducted a new pilot study [53], intended to examine the feasibility of stress management sessions during the ER stay with candidate interventions as selected by our literature search. To this end, we conducted a randomized open-label, single-center study to assess the feasibility of psychologist-led interventions in the context of the ER and to compare the effect of EMDR with reassurance and usual care. Conducted in the ER of Bordeaux University Hospital, the study included patients with a high risk of PCLS randomized into three groups, as follows: (1) a 15-min reassurance session, (2) a 60-min session of EMDR, and (3) usual care. Main outcomes were the proportion of interventions that could be carried out and the prevalence of PCLS and PTSD 3 months after the ER visit.

A total of 130 patients with a high risk of PCLS were randomized. No logistic problem or patient refusal was observed. In the EMDR, reassurance and control groups, the proportions of patients with PCLS at 3 months were 18%, 37%, and 65% and those with PTSD were 3%, 16%, and 19%, respectively. The relative risk for PCLS adjusted for the type of event (injury, non-injury) for the comparison between EMDR and control was 0.24 (95% CI 0.095–0.61). This first randomized controlled trial therefore shows that a short EMDR intervention is feasible and potentially effective in the context of the ER. The study was registered at ClinicalTrials.gov (NCT03194386).

The present protocol aims to replicate the latter trial in order to confirm or reject our hypothesis of a beneficial impact of early R-TEP EMDR on PCLS and PTSD in two different ERs. SPIRIT Checklist for this trial is provided as an Additional file 1.

### Potential benefit

The trial is designed to test the impact of early EMDR intervention on PCLS and PTSD in patients presenting to the ER. In 2012, the year of the last national survey in France, 10.6 million people attended the ER, some of whom several times, since 18 million visits were recorded that year. The SOFTER 3 study therefore addresses a major public health challenge.

## Methods/design

The main objective in our two-site, open-label, randomized controlled trial is to compare the impact on 3-month PCLS of early EMDR R-TEP intervention and usual care in patients presenting to the ER. Secondary objectives include the comparison between EMDR R-TEP and control of 3-month PTSD, 12-month PCLS, self-reported stress at ER discharge, self-assessed recovery expectation at discharge and 3 months, and self-reported pain at discharge and 3 months.

The outcomes are therefore defined as follows:

Primary outcome

- 3-month PCLS as measured with the Rivermead Post-Concussion Symptoms Questionnaire [54]
  Secondary outcomes
- 12-month PCLS as measured with the Rivermead Post-Concussion Symptoms Questionnaire
- 3-month PTSD as measured with PTSD Checklist-5 [55]
- Self-assessed recovery expectation at discharge and 3 months
- Self-reported chronic pain at 3 months
- Self-reported acute pain at discharge
- Psychotropic medicine use at 3 months as measured by drug delivery data extracted from the Caisse national d’assurance maladie des travailleurs salariés (CNAM-TS) database, the French social insurance system

### Randomization and blinding

Patients will be allocated to one of the two arms with block randomization by clinical center sites. Statistical analysis will be performed blinded to arm content, revealed only by the Data Safety Monitoring Board (DSMB) report. It is not possible to blind the participants to their allocation due to the nature of the intervention.

### Inclusion criteria

All patients attending the adult ER of one of the study sites following an event that led to an injury, or with a new acute medical condition, will be assessed for inclusion. The inclusion criteria are as follows:

- Age 18 and above
- Conscious, able to provide informed consent, able to understand study procedures and to comply with them for the entire length of the study; French speaker
- Injured, whatever the cause of injury (the event causing the injury must have occurred in the past 12 h) or experiencing a medical event associated with an acute medical condition and presenting for the first time to the ER for this reason
- Score resulting from the screening tool > 1: female: + 1, taking at least one anxiolytic treatment: + 1, perceived health status prior to admission: excellent, very good 0; good: + 1; poor: + 2; bad + 3
- Affiliated to the French insurance system

### Exclusion criteria

Any candidates to whom any of the exclusion criteria apply at baseline will be excluded from study participation. The exclusion criteria are as follows:

- Acute drug or alcohol use or dependence that, in the opinion of the site investigator, would interfere with adherence to study requirements
- Inability or unwillingness of individual or legal guardian/representative to give written informed consent
- Inability or unwillingness to be contacted for 3- and 12-month follow-up interviews

### Study enrollment procedures and randomization

**Fig. 1:**
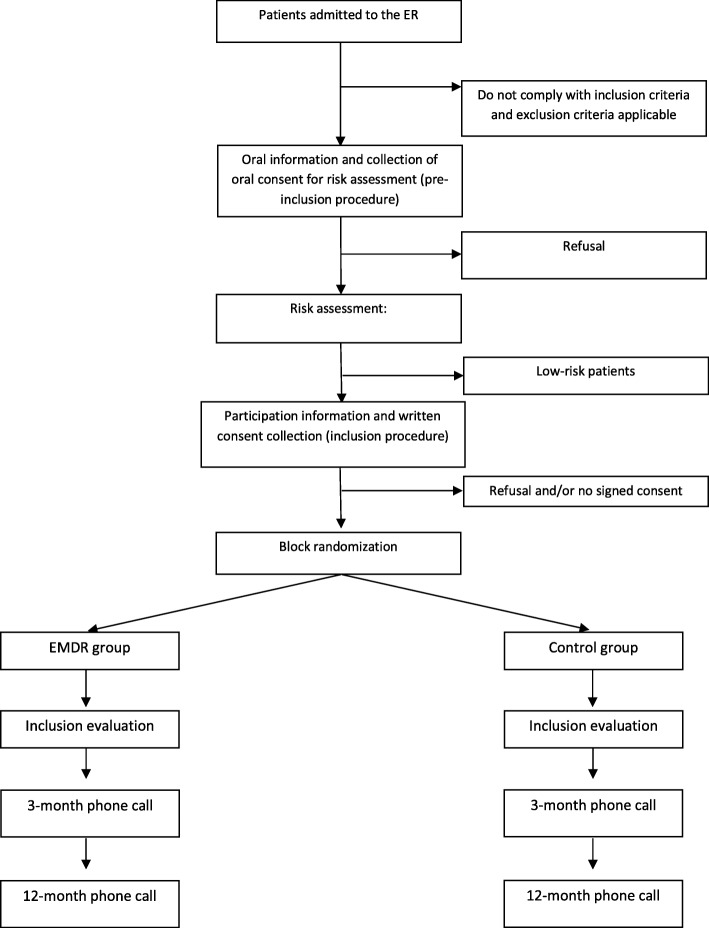
Overview study diagram

**Fig. 2:**
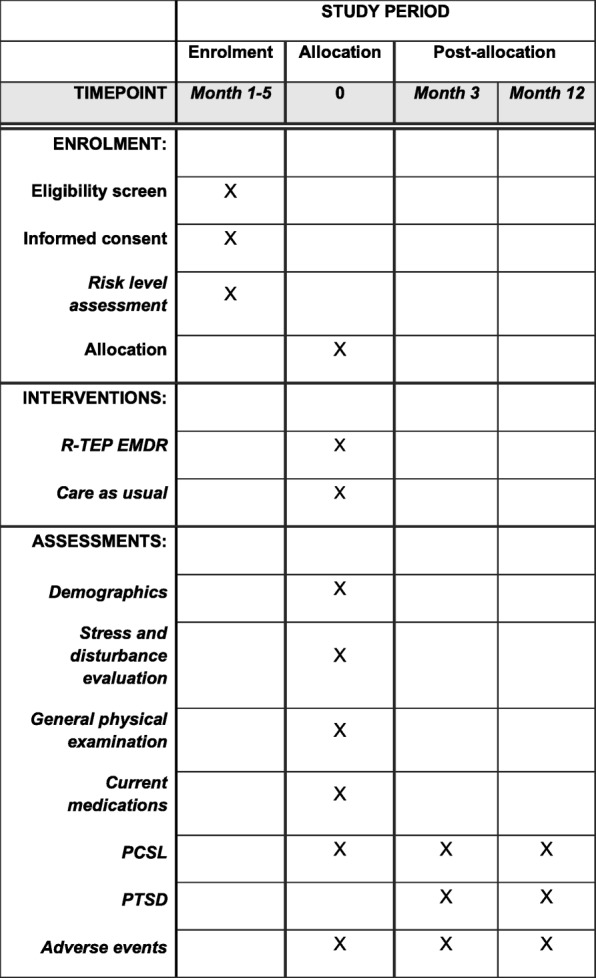
Schedule of enrolment, interventions, and assessments

Study protocol and time of collection of outcomes are presented in Figs. 1 and 2. Participants will be recruited among patients presenting to the ERs of the University Hospital of Bordeaux (Groupe Hospitalier Pellegrin) and Lyon (Groupement Hospitalier Edouard Herriot) and assessed with a high risk of PCLS. The identification and recruitment of potential study participants will be carried out by emergency personnel under the supervision of the project manager as soon as the patient’s condition permits and in all cases after the initial clinical evaluation conducted in the framework of the usual care. First oral consent will then be sought for participation in the assessment stage, which consists in selecting patients with a high risk of PCLS.

A set of three items will be recorded for each injured patient, including sex (+ 1 for female), perceived health status prior to admission (excellent, very good: 0; good: + 1 poor: + 2; bad: + 3), current use of anxiolytics/antidepressants (+ 1 if yes).

To be enrolled in the study, the patient will need to score above a pre-defined threshold of 2 on the scoring procedure based on the three items and designed to select patients at risk for PCLS. The score has been developed using data from the Pericles study and validated on data of the SOFTER Pilot 1 and 2 studies.

Patients fulfilling inclusion criteria and assessed as at risk for PCLS will be presented with the objective and procedures and invited to sign an informed consent form. A screening log will be filled in to describe reasons for ineligibility and for non-participation of eligible candidates.

The randomization procedure for assigning a participant to an intervention group will then be performed and the results will be recorded in the Shared Study Monitoring System. Electronic block randomization will be stratified according to study center. Block sizes will be randomly modified and kept secret.

### Intervention

Patients in the EMDR group will receive a 1-hour psychotherapeutic intervention, utilizing the R-TEP [40]. This protocol is specially designed for victims of recent traumatic events, and incorporates and extends the early EMDR intervention protocols [32] into an integrative and comprehensive intervention considering the fragmented, unconsolidated nature of recent traumatic memories and the need for safety and containment. Following the eight phases of the standard EMDR protocol, it introduces four new procedural concepts (Traumatic Episode, Episode Narrative, “Google Search/ Scan” for identifying disturbing fragments and Current Trauma Focused processing strategies). These sessions will be carried out by trained psychologists.

Patients in the treatment-as-usual group will be medically and psychologically attended to by ER staff with no intervention of the study psychologist.

### Sample size

The study sample size is calculated using PCLS rates expected at 3 months after an ER admission in a patient population assessed to be at high risk of PCLS.

Our pilot study showed that, using the criteria described above, the incidence of PCLS among patients selected and enrolled in the study will be of approximately 47%. Our aim is to design the present study to be able to evidence a 15% decrease in PCLS prevalence in the EMDR group. Assuming an alpha risk of 5% and a power of 80%, the required sample size will be 169 patients in each group. We further assumed 20% loss to follow-up and 5% missing data for the main variables. Thus, we plan to include 223 patients in each group (112 per center in each group).

A study therapist will be available from 10 am to 6 pm, 5 days a week. Considering EMDR session duration and emergency care, patients will be assessed for eligibility from 8 am to 6 pm. Data from our ER registry and experience from our pilot study show that, during this period, approximately 50 patients will be assessed for eligibility. The screening tool used in this study will select approximately 10% of patients admitted to the ER. We also estimate that 10% of eligible patients will be missed in the ER and assume a 5% refusal rate. Consequently, we can expect approximately four inclusions per day, corresponding to an inclusion period of 3 months.

### Adherence assessment

Adherence to the study regimen will be defined as the extent to which participants comply with study intervention requirements. The SOFTER Pilot study 2 showed that over 95% adherence can be expected in the EMDR group. A log of intervention sessions will be kept for each participant and will include duration, completeness, and patient satisfaction. This log will be regularly reviewed by the Steering Committee and used as part of the decision to continue or discontinue the study.

### Interim analyses and stopping rules

No interim analysis of efficacy is planned. The study can be stopped by the DSMB for safety reasons or because of poor study performance (losses to follow-up > 25%), poor quality control, slow accrual (recruitment rate < 75% than expected), serious adverse events considered to be caused by the intervention, or increased frequency of adverse events. Such findings are presented to the DSMB for review of the events to determine whether there are statistical as well as clinical concerns. The statistician reports their findings to a closed session of the DSMB and these are used to determine what steps will be taken.

### Data analyses

Descriptive and inferential statistical methods will be used to analyze the outcomes and other study data. Confounding variables will include cause of admission (injury versus medical), age group, and sex. The analyses will be conducted as intent-to-treat for primary endpoint and per-protocol for secondary analyses. Randomization codes will only be revealed at the end of the analysis.

Primary analyses will be conducted using a Fisher exact test. A stratified analysis will be carried out considering study center and PCLS risk score. For other variables, Wilcoxon test will assess differences for continuous variables and Fisher exact test for categorical variables.

Differences between patients who completed the study and those who were lost to follow-up will be assessed for all variables.

### Dissemination

The results of the trial will be published regardless of the direction of effect. Communications will be presented at specialized conferences and reports will be submitted to peer-reviewed medical journals.

### Quality control

A clinical research associate mandated by the sponsor will regularly visit each study center, when the research is set up, once or several times during the course of research, according to the rhythm of the inclusions and at the end of the research. During these visits, and in accordance with the monitoring plan, the following will be reviewed:

#### Informed consent

- Respect of the research protocol and procedures defined in it
- Quality of the data collected in the report file: completeness, accuracy, missing data, consistency of data with source documents (medical records, appointment books, original laboratory results, etc.)

All visits will be subject to a written monitoring report.

### Confidentiality of data

In accordance with the statutory provisions in place (the French Public Health Code), persons having direct access to source data will take every precaution required to ensure the confidentiality of information relating to investigational medicinal products, studies, and participants, notably concerning their identity, as well as the results obtained. These persons, like the investigators themselves, are subject to professional confidentiality.

During the clinical study or at its conclusion, data regarding participants that is collected and sent to the sponsor by the investigators (or all other specialists involved) will be anonymized. At no point will the names of participants or their addresses appear unencrypted.

Only the first letters of the first name and full name of included patients will be recorded, followed by a specific research number indicating the rank of inclusion and the origin of the investigator site.

The sponsor will ensure that each study participant has given their consent for access to their personal data, which is strictly required for study quality control.

### Data and Safety Monitoring Board (DSMB)

The DSMB is an independent group of experts that advises the study investigators. The members of the DSMB serve in an individual capacity and provide their expertise and recommendations. The primary responsibilities of the DSMB are to (1) periodically review and evaluate the accumulated study data for participant safety, study conduct and progress, and, when appropriate, efficacy, and (2) make recommendations concerning the continuation, modification, or termination of the trial. The DSMB considers study-specific data as well as relevant background knowledge about the patient population under study.

The DSMB is responsible for defining its deliberative processes, including event triggers that would call for an unscheduled review, stopping guidelines, unmasking, and voting procedures prior to initiating any data review.

The study DSMB consists of three independent experts, inclduing one expert in the clinical aspects of the stressed/injured patient population; one biostatistician with expertise in current clinical trial conduct and methodology; and one expert in psychotherapeutic EMDR interventions.

The DSMB has been appointed prior to study initiation.

### Premature withdrawal from the study and withdrawal of consent

The participant has the right to withdraw from the research at any time. If participants decide to withdraw from all components of the study, they are no longer followed up in the protocol. Premature withdrawal from the research strategy must be notified promptly to the Steering Committee. The reasons for and the date of withdrawal must be documented. The withdrawal of consent is a decision by a participant to reconsider their decision to participate in the research and to assert their right to withdraw consent at any time during follow-up, without resulting in any prejudice thereby and without having to justify it. When a participant withdraws consent for participation in the research, data already collected for this patient will be kept for analysis.

### Protocol deviations

Deviations can affect all aspects of a research protocol such as inclusion, monitoring, measurement of endpoints, and treatment process. All deviations must be documented by the investigator and discussed by the Steering Committee and Data Management Center.

Even in the event of deviation from the protocol, participants must be monitored until the date planned in the protocol.

### Archiving study documents and study data

The protocol and any changes to the protocol, report files (copies), source files of participants who gave consent, and all other documents and correspondence related to the research will be archived in accordance with good clinical practices for a period of 15 years following the end of the research. The original informed consent forms of participants will be archived for a period of 30 years following the end of the research.

### Ethical approval

The sponsor and the investigator(s) undertake the responsibility to ensure that the research is conducted in compliance with Law no. 2012–300 on research involving human participants of 5 March 2012, in accordance with Good Clinical Practices (I.C.H. version 4 of 9 November 2016 and Decision of 24 November 2006), and the Declaration of Helsinki.

The research will be conducted in accordance with the present protocol. Except in emergency situations requiring specific medical procedures, the investigator undertakes the responsibility to comply with the protocol in all respects, particularly with regard to the collection of consent, and the reporting and monitoring of serious adverse events.

This research project has received positive endorsement from the French CPP (Comité de protection de Personnes Ouest II - Angers). N° RCB = 2017-A01462–51 – N°CPP = 2017/36.

The University Hospital of Bordeaux, the sponsor of this research, has taken out a civil liability insurance contract with Gerling-Biomedicinsure in accordance with the provisions of the public health code.

The data recorded in the course of this research shall be subject to computer processing on behalf of INSERM U1219 Bordeaux Population Health Research Center in compliance with Law No. 78–17 of 6 January 1978 relating to data processing, files and freedoms, as amended by Law 2004–801 of 6 August 2004.

This research project falls within the framework of the “Reference Methodology” (MR-001) in application of the provisions of article 54, paragraph 5 of the amended law of 6 January 1978 relating to information, files and freedoms. This change was approved by the decision of 5 January 2006, updated on 21 July 2016. The INSERM U1219 Bordeaux Population Health Research Center has signed a commitment to comply with this “Reference Methodology”.

A specific request for clearance will be submitted to the Commission Nationale Informatique et Liberté (CNIL) in order to obtain the authorization to use the national social security ID to retrieve medication data at 3 and 12 months.

## Discussion

The trial is designed to test the impact of early EMDR intervention on PCLS and PTSD in patients presenting to the ER. In 2012, the year when the last national survey in France was undertaken, 10.6 million people attended the ER, some of whom several times, since 18 million visits were recorded that year. The SOFTER 3 study therefore addresses a major public health challenge.

We already described the feasibility of short EMDR sessions in the ED during the SOFTER 2 study [53], which also found a superiority of EMDR versus reassurance versus control. We need to confirm these results in a larger and more diverse population.

## Trial status

The present publication refers to the 4.0 version of the SOFTER 3 protocol dates on 01/02/2018. Recruitment began on January 15, 2018, and is expected to be completed by the June 15, 2018.

## Additional file

Additional file 1: SPIRIT Checklist (DOC 121 kb)

## Abbreviations

DSMB: Data Safety Monitoring Board
EMDR: eye movement desensitization and reprocessing
ER: emergency room
ERP: emergency response procedure
PCLS: post-concussion-like symptoms
PCS: post-concussion syndrome
PTSD: post-traumatic stress disorder
R-TEP: recent traumatic episode protocol
SOFTER: SymptOms Following Trauma, Early Response
